# Current state of endovascular treatment of anterior cerebral artery aneurysms

**DOI:** 10.3389/fneur.2024.1396701

**Published:** 2024-07-30

**Authors:** Bingwei Li, Kun Zhang, Jinlu Yu

**Affiliations:** ^1^Department of Neurosurgery, First Hospital of Jilin University, Changchun, China; ^2^Department of Cerebrovascular Disease, Henan Provincial People's Hospital of Zhengzhou University, Zhengzhou, China

**Keywords:** anterior cerebral artery, aneurysm, endovascular treatment, prognosis, complication, review

## Abstract

The locations of anterior cerebral artery (ACA) aneurysms vary, and various aneurysms can occur along the course of the ACA. Ruptured and some unruptured ACA aneurysms may require aggressive treatment to avoid bleeding or rebleeding. Although open surgery is an effective treatment for ACA aneurysms, endovascular treatment (EVT) is becoming an alternative treatment in select cases. EVT techniques for ACA aneurysms often vary and are performed on a case-by-case basis according to the nature and location of the aneurysm. To better understand the EVT strategy for ACA aneurysms, it is necessary to review EVT for ACA aneurysms. In this review, the following topics are discussed: ACA anatomy and anomalies, classifications of ACA aneurysms, the natural history of ACA aneurysms, open surgery and EVT statuses for ACA aneurysms, EVT techniques for various ACA aneurysms, and the prognosis and complications of EVT for ACA aneurysms. According to our review and experience, traditional coiling EVT is still the preferred therapy for most ACA aneurysms. For A1 aneurysms, EVT is challenging. After the selection of appropriate cases, deployment of a flow diverter and Woven EndoBridge device can result in a good prognosis for patients with ACA aneurysms. In addition, parent artery occlusion can be used to treat A1 aneurysms with good collateral circulation and some distal ACA aneurysms. In general, EVT is gaining popularity as an alternative treatment option for ACA aneurysms.

## 1 Introduction

The anterior cerebral artery (ACA) is a complex artery system. Along its course, the ACA splits into numerous perforating arteries and cortical branches (1). Various locations and types of aneurysms can occur along the ACA (2). Currently, both open surgery and endovascular treatment (EVT) can be used as treatment options for ACA aneurysms. However, there has been a transition from open surgery to EVT as the first choice (3). Although EVT to treat ACA aneurysms is feasible, the EVT technique is challenging.

Various EVT techniques, including traditional coiling with/without balloon or stent assistance, deployment of a flow diverter (FD) and Woven EndoBridge (WEB) device (Sequent Medical, Aliso Viejo, California), and parent artery occlusion (PAO), can be used in EVT for ACA aneurysms (4–6). Because EVT techniques for ACA aneurysms vary and are performed on a case-by-case basis according to the nature and location of the aneurysm, EVT experiences need to be summarized. To better understand the strategy of EVT for ACA aneurysms, a complete review is necessary.

## 2 ACA anatomy and anomalies

**Figure 1:**
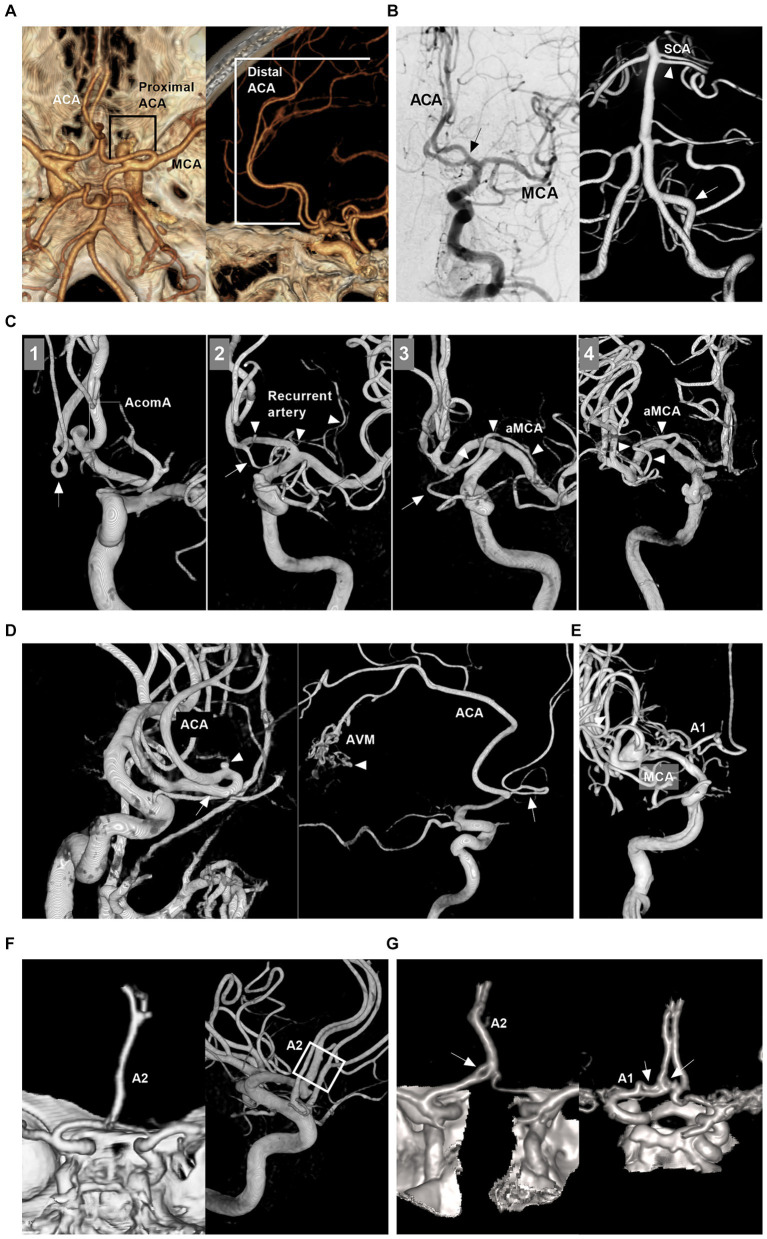
Intracranial ACA anatomy and anomalies. **(A)** CTA of superior to inferior (left panel) and lateral (right panel) views showing proximal and distal segments of the ACA. **(B)** Left panel: DSA of the ICA showing the early bifurcation (arrow) of the ACA at A1 segment; Right panel: 3D-DSA of the VA showing a large fenestration (arrow) of the VA and double SCAs (arrowhead); Images of left and right panels were from the same patient. **(C)** Panel 1: 3D-DSA of ICA showing the cortical branch from the AcomA; Panel 2: 3D-DSA of ICA showing that the recurrent artery of Heubner (arrowheads) and cortical artery (arrow) shared a common trunk from distal A1 segment; Panel 3: 3D-DSA of ICA showing that the aMCA (arrowheads) and cortical artery (arrow) shared a common trunk from distal the A1 segment. Panel 4: 3D-DSA of ICA showing that the aMCA (arrowheads) from the A1 segment near its origin. **(D)** Left panel: 3D-DSA of ICA showing proximal ACA course along the olfactory nerve (arrow) with an aneurysm (arrowhead) on it; Right panel: 3D-DSA of ICA showing the persistent olfactory artery (arrow) and an AVM (arrowhead). **(E)** 3D-DSA of ICA showing that the ACA originated from the lenticulostriate artery. **(F)** Left panel: CTA showing the azygos ACA (arrow); Right panel: 3D-DSA of ICA showing the triplication of ACA (frame). **(G)** Left panel: CTA showing a fenestration (arrow) of the A1 and the azygos ACA; Right panel: CTA showing two fenestrations (arrows) of A1 and AcomA. A1 and A2, first and second segments; ACA, anterior cerebral artery; AcomA, anterior communicating artery; aMCA, accessory middle cerebral artery; AVM, arteriovenous malformation; 3D, three-dimensional; CTA, computed tomography angiography; DSA, digital subtracted angiography; ICA, internal carotid artery; SCA, superior cerebellar artery; VA, vertebral artery.

The ACA is divided into proximal (A1) and distal (A2-A5) segments by the anterior communicating artery (AcomA) (Figures 1A,B) (1). Together with the callosomarginal artery, the pericallosal artery branches off into the cortical arteries. Some small cortical arteries can originate from the A1 segment and AcomA (Figure 1C) (7). The A1 segment, AcomA segment and proximal A2 segment branch off into numerous perforating arteries, of which the recurrent artery of Heubner is one of the largest (Figure 1C) (1, 7).

The ACA can exhibit many anomalies. It may originate from the cavernous internal carotid artery (ICA), ophthalmic artery, or even the MCA and lies under the optic nerve or along the olfactory nerve (persistent olfactory artery) (Figure 1D) (8). A1 may be duplicated, asymmetric or absent (Figure 1E) (8). AcomA may be plexiform, duplicated or absent (9). Distal ACA anomalies include azygos ACA, bihemispheric A2 or A3 branches to the contralateral hemisphere, or triplication (Figure 1F) (9). Fenestration can occur at the ACA (Figure 1G). An accessory MCA can originate from A1, AcomA or proximal A2 (Figure 1C) (10). Pure arterial malformation can also occur on the ACA (11).

## 3 Classifications of ACA aneurysms

### 3.1 Morphology and pathology

**Figure 2:**
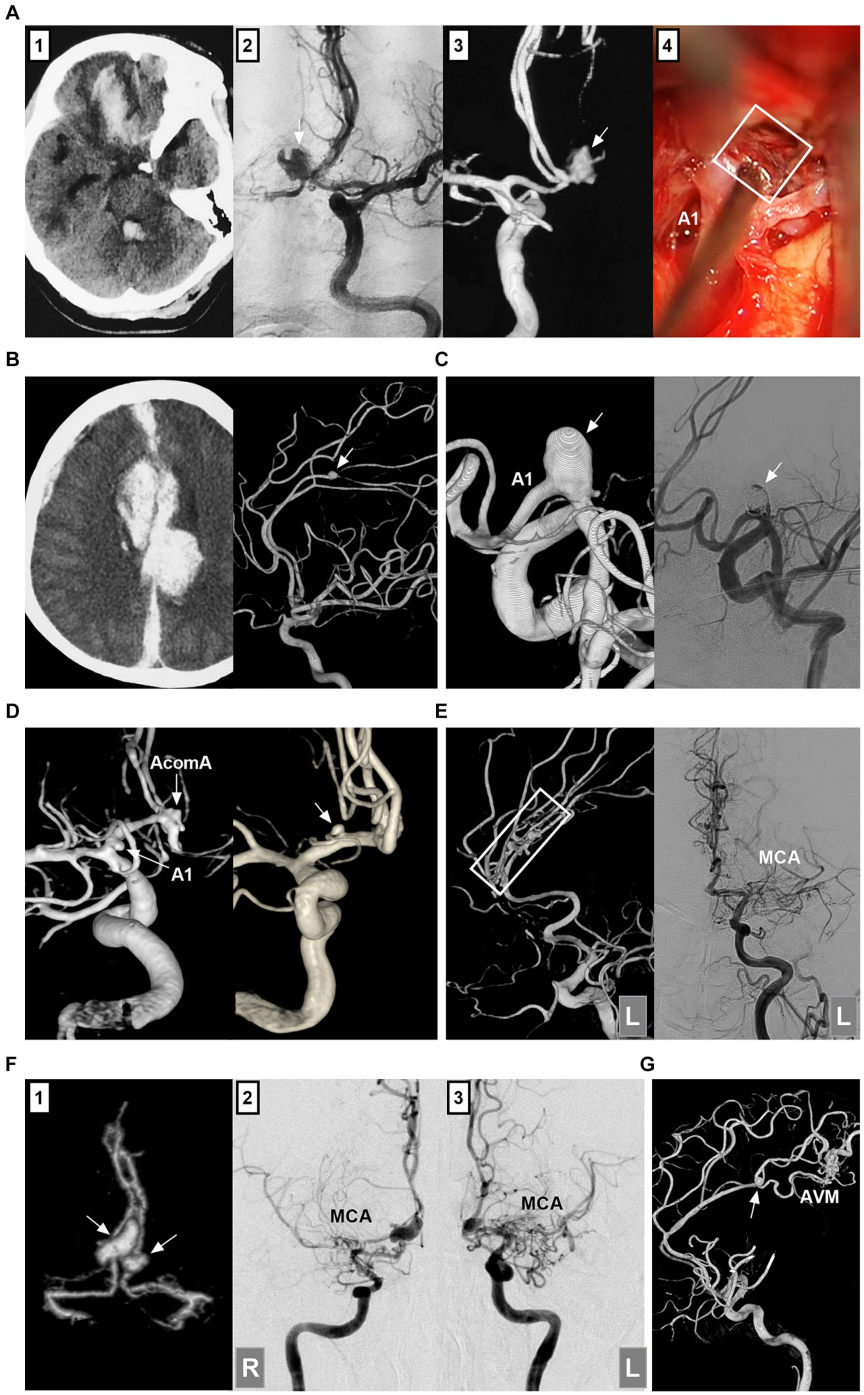
Classifications of ACA aneurysms. **(A)** Panel 1: CT showing contusion in the frontal lobe and hemorrhage in the fourth ventricle; Panels 2 and 3: DSA showing the traumatic pseudoaneurysm (arrows) at the AcomA; Panel 4: Intraoperative image showing the pseudoaneurysm (frame). **(B)** Left panel: CT showing traumatic hematoma in the longitudinal fissure and left subdural hematoma. Right panel: 3D-DSA showing the traumatic pseudoaneurysm (arrow) at the distal ACA. **(C)** Left panel: 3D-DSA showing a dissecting aneurysm in the A1 origin; Right panel: DSA showing the aneurysm after coiling. **(D)** Left panel: 3D-DSA showing an A1 aneurysm (arrow) and an AcomA aneurysm (arrow); Right panel: 3D-DSA showing an aneurysm (arrow) at the origin of the recurrent artery of Heubner. **(E)** Left panel: 3D-DSA of the left ICA showing multiple aneurysms (frame) at the distal ACA; Right panel: DSA of left ICA showing the occluded MCA. **(F)** Panel 1: CTA showing mirror-like distal ACA aneurysms (arrows); Panels 2 and 3: DSA of ICAs showing bilateral twig-like MCAs. **(G)** 3D-DSA showing an AVM with a flow-related distal ACA aneurysm (arrow). 3D, three-dimensional; A1, first segment; ACA, anterior cerebral artery; AcomA, anterior communicating artery; AVM, arteriovenous malformation; CT, computed tomography; CTA, CT angiography; DSA, digital subtracted angiography; ICA, internal carotid artery; L, left; MCA, middle cerebral artery; R, right.

ACA aneurysms can be divided into saccular and nonsaccular lesions according to their morphology and pathology (12). Most ACA aneurysms are saccular lesions (13). Nonsaccular lesions may be caused by dissection, trauma, infection, etc. (Figures 2A–C) (14–16). Traumatic or infectious aneurysms are often false (pseudoaneurysms) and exhibit severe arterial wall disruption, even with adventitia perforation (17).

### 3.2 Location

ACA aneurysms can be divided into proximal A1, AcomA and distal A2--A5 lesions (Figures 2D,E) (18). A1 aneurysms are rare and often small saccular lesions located at the origin of the perforating artery or A1 fenestration (19). A1 dissection or pseudoaneurysm is rare (16). Aneurysms rarely occur at the origin of the recurrent artery of Heubner and at the origin of the accessory MCA from the ACA (20).

The AcomA region is one of the most common locations for aneurysms because of hemodynamic force alterations caused by aplasia or hypoplasia of the A1 segment (21). Distal ACA aneurysms are uncommon, with most located at the junction of the ACA trunk and major cortical branch and fewer located at the peripheral cortical branch (22). Most distal lesions are saccular (23). Distal ACA aneurysms can be dissected or pseudoaneurysms, which tend to occur at the A2 segment (13, 24).

### 3.3 Other classifications

According to the International Subarachnoid Aneurysm Trial (ISAT), ACA aneurysms can be divided into small (<7 mm), medium (7–12 mm), large (>12–25 mm), or giant (>25 mm) lesions (25). Small aneurysms ≤ (3 mm) were defined as tiny ACA aneurysms (26). Most ACA aneurysms are small or medium-sized (27). Giant ACA aneurysms are rare (28). ACA aneurysms can be tandem or mirror-like, or they can be associated with aneurysms at other locations (Figure 2F) (29, 30). ACA aneurysms may be flow-related and are associated with brain arteriovenous malformation (AVM), moyamoya disease (MMD), twig-like MCA, and MCA or ICA occlusion (Figures 2E–G) (31–33).

## 4 Natural history of ACA aneurysms

The natural history of ACA aneurysms varies depending on the ruptured or unruptured state, size, and saccular and nonsaccular types.

### 4.1 Saccular aneurysms

Untreated ruptured intracranial aneurysms have a poor natural history. In Korja et al.’s report on untreated ruptured intracranial aneurysms in 510 patients, nearly 80% were located in the anterior circulation. After conservative treatment, the 1-year mortality rate was 65%, the 5-year mortality rate was 69%, and the 10-year mortality rate was 76%; hospital-admitted poor-grade patients who were untreated had 1-year mortality rates of approximately 90% (34). The natural history of ruptured ACA aneurysms should be similar to that in the above report. In Nishioka et al.’s report, mortality rates for untreated ruptured AcomA aneurysms at 3 months, 6 months, 1 year and 2 years were 39, 40, 41 and 42%, respectively; for distal ACA aneurysms, mortality rates at 3 months, 6 months, 1 year and 2 years were 55, 60, 65 and 65%, respectively (35).

Unruptured ACA aneurysms are associated with increased rupture risk (36). In Clarke et al.’s review of 228 unruptured ACA aneurysms, including aneurysms in the ACA, AcomA, and pericallosal artery, the overall annual rupture rate was 1.7% (37). In Mira et al.’s meta-analysis, unruptured AcomA aneurysms presented a risk of rupture that was twice as high as that of other aneurysms (38). Takeda et al. reported that distal ACA aneurysms may rupture, even when they are small (39). In the natural history of unruptured ACA aneurysms, size plays an important role, and the rupture rate increases with size (28, 40). In the Japanese Unruptured Cerebral Aneurysm Study (UCAS), the rates of rupture per aneurysm per year for AcomA aneurysms were 0.9, 0.75, 1.97, 5.24 and 39.77% for 3–4 mm, 5–6 mm, 7–9 mm, 10–24 mm and ≥ 25 mm aneurysms, respectively (41).

### 4.2 Nonsaccular aneurysms

The natural history of nonsaccular ACA aneurysms is unclear. Unruptured lesions can be stable (12). For small traumatic and ruptured dissecting ACA aneurysms with fewer tears, spontaneous repair of the arterial wall by collagen proliferation can be expected starting from the first week, and healing becomes effective after 4–5 weeks (42). However, for most nonsaccular ruptured ACA aneurysms, the rebleeding risk is high, especially in the first month after onset (13, 43, 44). For flow-related ACA aneurysms with brain AVM, MMD or MCA/ICA occlusion, the natural history is unclear. Owing to hemodynamic stress, these patients may have a worse natural history than those with no associated lesions (32, 45).

## 5 Open surgery and EVT statuses

For ruptured or unruptured growing, giant, or symptomatic ACA aneurysms, treatment can be considered. Open surgery and EVT can be used for ACA aneurysms. The decision should be made on a selective, case-by-case basis to maximize patient benefits and limit the risk of periprocedural complications.

For A1 aneurysms, open surgery is still the first choice (46). Open surgery easily exposes A1 aneurysms. During surgery, perforating arteries, the recurrent artery of Heubner, and medial striate arteries might be clearly identified and preserved; in addition, the dissection range for A1 aneurysms can be identified (47). EVT can only be performed for select A1 aneurysms. For AcomA aneurysms and distal ACA aneurysms, there are no standard clinical decisions regarding open surgery or EVT. Both modalities are effective in preventing aneurysm rupture after contrastive analysis (21, 48–50). For distal ACA aneurysms, open surgery is associated with greater aneurysm occlusion and lower recurrence (51). However, owing to new equipment, such as the low-profile Neuroform Atlas stent (Stryker Neurovascular, Fremont, California, USA) and small FD, EVT has become increasingly common for AcomA aneurysms and distal ACA aneurysms (18, 52).

## 6 EVT techniques for various ACA aneurysms

EVT options for ACA aneurysms include reconstructive treatment with traditional coiling EVT with/without a stent or balloon assistance, FD deployment to preserve the ACA, a WEB device or PAO.

### 6.1 Proximal ACA aneurysms

**Figure 3:**
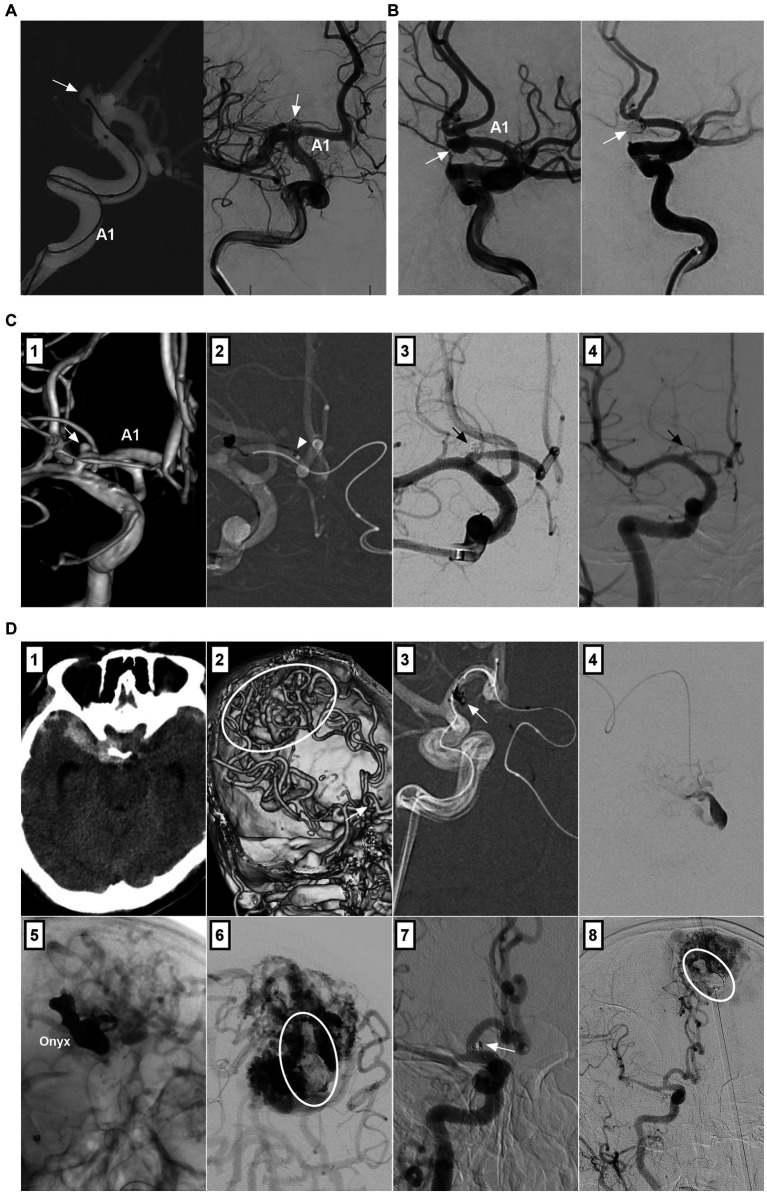
Treatment of A1 aneurysms by traditional coiling. **(A)** Left panel: Roadmap image showing the straight microcatheter inserted into the A1 aneurysm (arrow); Right panel: DSA showing the A1 aneurysm (arrow) after coiling. **(B)** Left panel: DSA showing an aneurysm (arrow) at A1 termination; Right panel: DSA showing the aneurysm (arrow) after coiling. **(C)** Panel 1: 3D-DSA showing an A1 aneurysm (arrow); Panel 2: Roadmap image showing the A1 aneurysm coiled via contralateral approach; the arrowhead indicates the distal marker of the stent; Panel 3: Postoperative immediate DSA showing the A1 aneurysm (arrow) after coiling; Panel 4: Six-month follow-up DSA showing that the A1 aneurysm (arrow) had no recurrence. **(D)** Panel 1: CT showing subarachnoid hemorrhage; Panel 2: CTA showing an aneurysm (arrow) at A1 segment and an AVM (circle); Panel 3: Roadmap image showing the A1 aneurysm (arrow) coiled via contralateral approach; Panel 4: Selective microcatheter angiography showing the aneurysmal structure in the AVM; Panels 5 and 6: DSA showing Onyx casting (circle) in aneurysmal structure; Panel 7: Six-month follow-up DSA showing that the A1 aneurysm (arrow) had no recurrence; Panel 8: DSA showing no recurrence of the aneurysmal structure in the AVM (circle). 3D, three-dimensional; A1, first segment; ACA, anterior cerebral artery; AVM, arteriovenous malformation; CT, computed tomography; CTA, computed tomography angiography; DSA, digital subtracted angiography.

Because the origin of perforating arteries usually arises from the superior and/or posterior wall of the A1 segment, A1 saccular aneurysms are often small and project upward or backward, and there is a sudden directional turn of A1 from the ICA, which makes it difficult for the microcatheter to access the aneurysm (53). Fenestration increases the difficulty of EVT (19). EVT can only be performed in A1 saccular aneurysms with easy catheterization, such as those located at the beginning and termination of A1 (Figures 3A,B). When an ipsilateral approach fails, a contralateral approach via the AcomA can be used to access A1 aneurysms if a competent AcomA exists (Figures 3C,D) (54).

**Figure 4:**
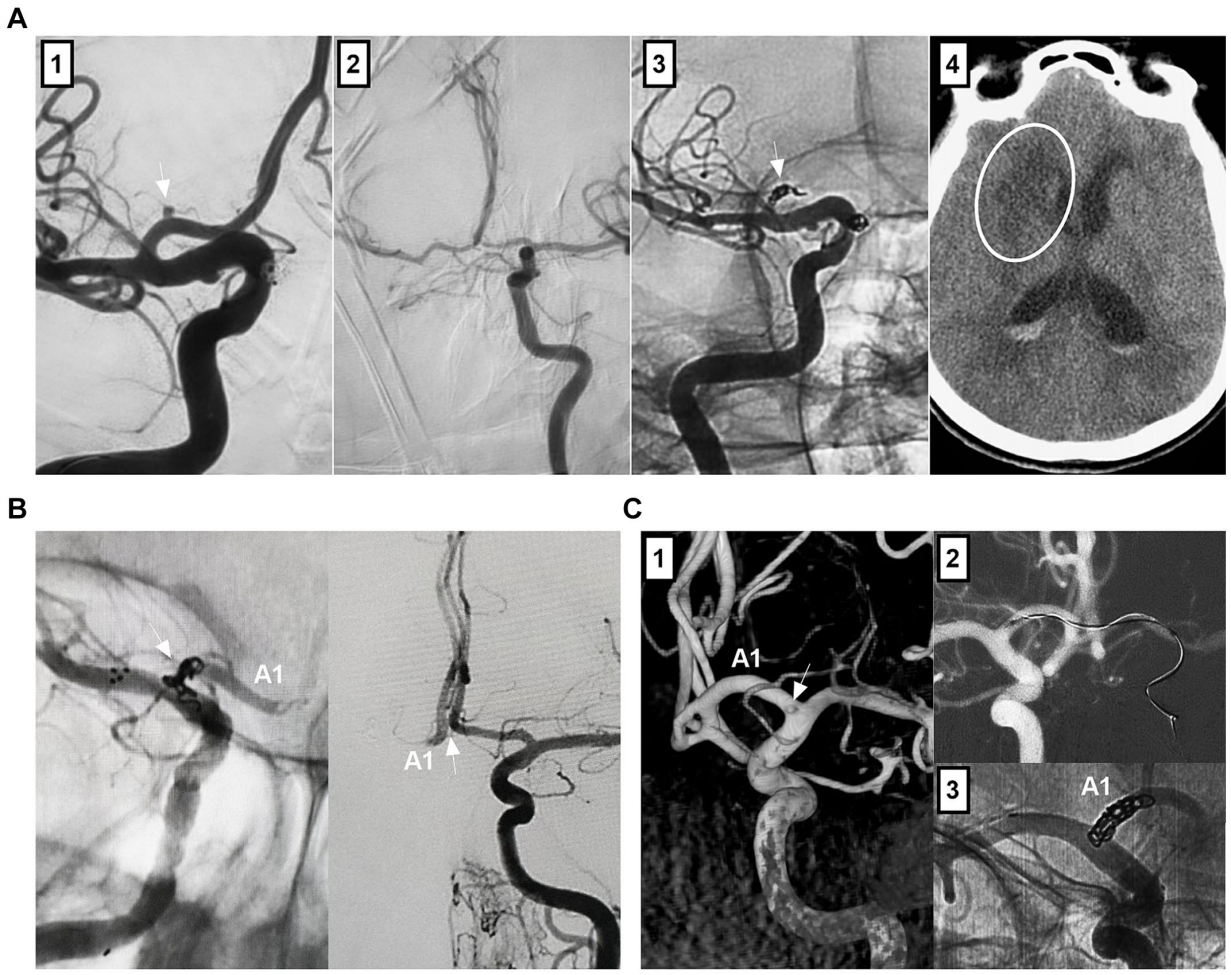
Treatment of A1 aneurysms by parent artery occlusion. **(A)** Panel 1: DSA showing an A1 aneurysm (arrow); Panel 2: DSA showing the compensation of the blood flow from contralateral ICA via the competent AcomA; Panel 3: Unsubtracted DSA showing the coiling (arrow) of A1 segment; Panel 4: Postoperative CT showing the infarction (circle) of head of caudate nucleus. **(B)** Left panel: Unsubtracted DSA showing that A1 aneurysm was coiled together with the A1 origin (arrow) by ipsilateral ICA approach; Right panel: DSA of contralateral internal carotid artery showing the patent AcomA (arrow). **(C)** Panel 1: 3D-DSA showing that a tiny A1 aneurysm; Panel 2: Roadmap image showing the contralateral approach to occlude the A1 aneurysm; Panel 3: Unsubtracted DSA showing that A1 aneurysm was coiled together with the A1 origin. 3D, three-dimensional; A1, first segment; ACA, anterior cerebral artery; AcomA, anterior communicating artery; CT, computed tomography; DSA, digital subtracted angiography; ICA, internal carotid artery.

For nonsaccular A1 aneurysms, when there is a competent AcomA and the contralateral A1 fills the bilateral ACAs, PAO can be performed to trap the aneurysm (Figures 4A–C) (24). However, PAO was associated with A1 perforating artery occlusion (Figure 4A) (47). When the contralateral A1 is hypoplastic or when cross flow through the AcomA is deficient, reconstructive EVT to preserve A1 must be performed.

**Figure 5:**
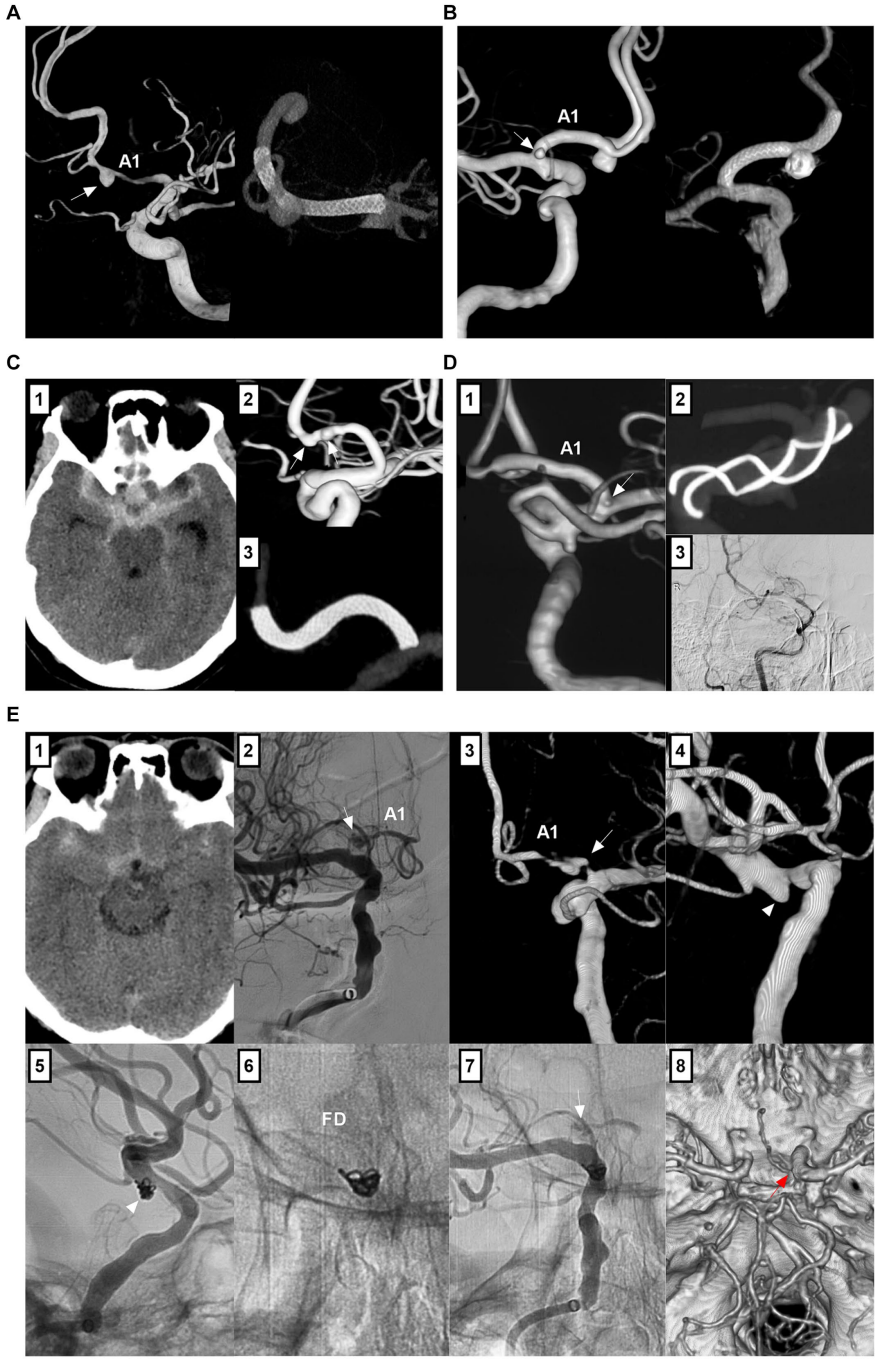
Treatment of A1 aneurysms by FD deployment. **(A)** Left panel: 3D-DSA showing an A1 dissecting aneurysm (arrow); Right panel: Vaso-reconstructive image showing the FD covering the aneurysm. **(B)** Left panel: 3D-DSA showing tandem A1 dissecting (arrow) and AcomA aneurysms; Right panel: Vaso-reconstructive image showing the FD covering these two aneurysms, the AcomA aneurysm was coiled. **(C)** Panel 1: CT showing subarachnoid hemorrhage; Panel 2: 3D-DSA showing two tandem A1 dissections (arrows); Panel 3: Vaso-reconstructive DSA showing the FD covering the two dissections. **(D)** Panel 1: 3D-DSA showing an aneurysm of posterior communicating artery and an A1 aneurysm (arrow) at its origin; Panel 2: Vaso-reconstructive DSA showing the FD deployment covered A1 origin; Panel 3: Six-month follow-up DSA showing the vessel stenosis in the FD. **(E)** Panel 1: CT showing subarachnoid hemorrhage; DSA (panel 2) and 3D-DSA (panel 3) showing the A1 origin dissecting aneurysm (arrows); Panel 4: 3D-DSA showing associated posterior communicating artery aneurysm (arrow); Panel 5: Unsubtracted DSA showing the coiled posterior communicating artery aneurysm (arrow); Panel 6: X-ray film showing that the FD covered the A1 origin; Panel 7: Unsubtracted DSA showing the decreased blood flow filled in the A1 dissection (arrow); Panel 8: Postoperative next day CTA showing that the A1 dissecting aneurysm (arrow) cannot be seen clearly. 3D, three-dimensional; A1, first segment; ACA, anterior cerebral artery; AcomA, anterior communicating artery; CT, computed tomography; CTA, computed tomography angiography; DSA, digital subtracted angiography; FD, flow diverter; ICA, internal carotid artery.

Owing to the dissecting or false nature of nonsaccular A1 aneurysms, simple coiling or coiling assisted by traditional stents with a 5–15% mental coverage rate may be an insufficient cure (24). FDs with a > 30% mental coverage rate alone or with adjunctive coiling can be used for unruptured A1 aneurysms (Figures 5A,B) (55). FD treatment for ruptured A1 aneurysms can be used because of the strong flow diversion effect, where primary coiling may not be technically feasible, such as in blister-like or tiny aneurysms (Figure 5C) (56). However, FD causes rebleeding in ruptured A1 aneurysms because aneurysm cure requires time, and antiplatelet agents can increase hemorrhagic risk (57). In addition to FD deployment in the A1 segment, in select cases, FD can be deployed from the MCA to the ICA, covering the ACA ostia to promote A1 aneurysm thrombosis (Figures 5D,E). For this method, the A1 aneurysm should be close to the ACA origin, and the contralateral A1 supplies the bilateral ACA; aneurysm occlusion is associated with ACA stenosis or occlusion (58).

### 6.2 AcomA aneurysms

**Figure 6:**
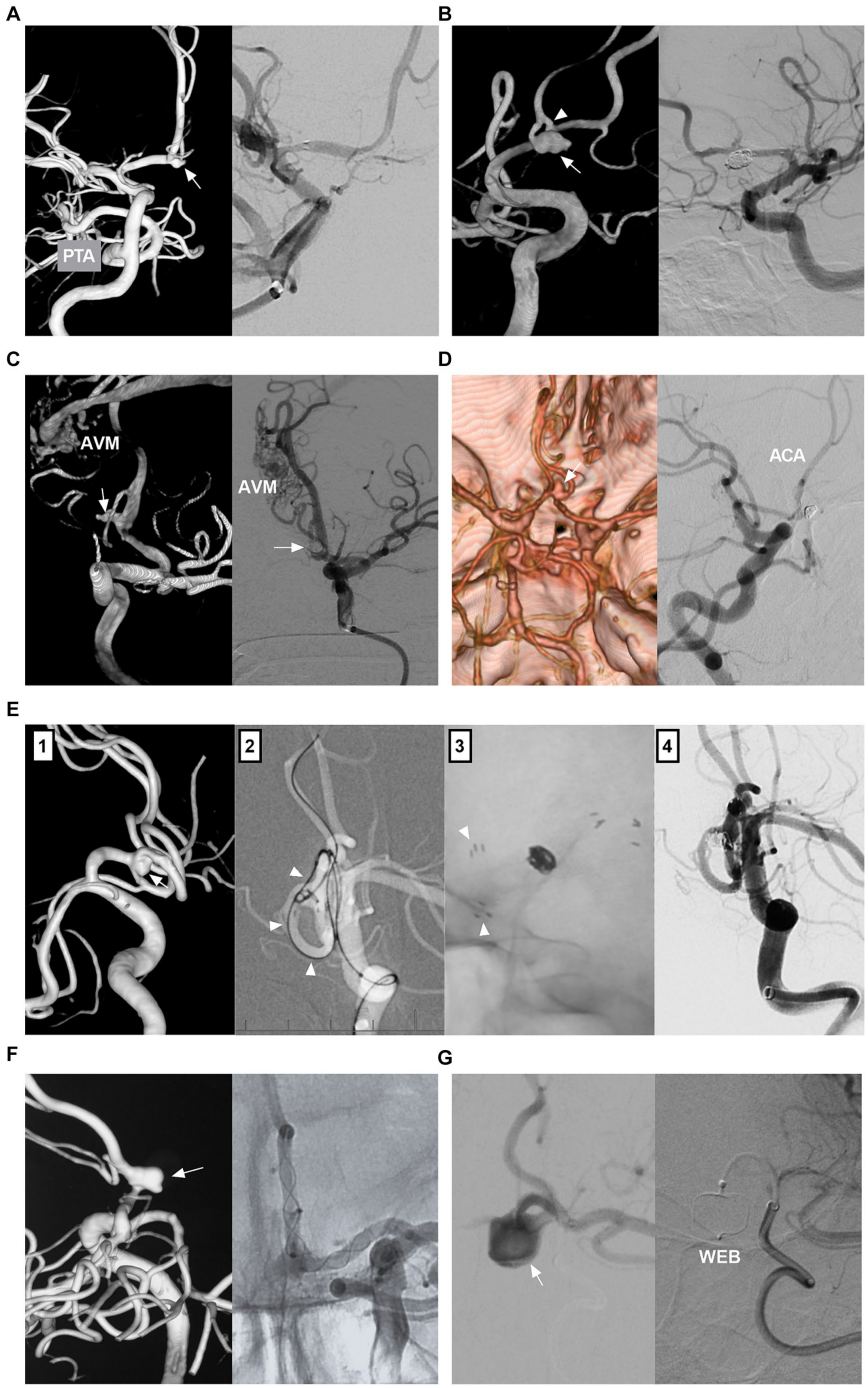
Treatment of AcomA aneurysms. **(A)** Left panel: 3D-DSA showing an AcomA aneurysm (arrow) with PTA; Right panel: DSA showing the coiled aneurysm with stent assistance. **(B)** Left panel: 3D-DSA showing an AcomA aneurysm (arrow) in a fenestration (arrowhead). Right panel: Six-month follow-up DSA showing that the aneurysm was occluded completely. **(C)** Left panel: 3D-DSA showing an AcomA aneurysm (arrow) and an AVM; Right panel: DSA showing the coiled aneurysm with the AVM intact. **(D)** Left panel: CTA showing an AcomA aneurysm (arrow) with symmetric ACAs; Right panel: Six-month follow-up DSA showing that the aneurysm was coiled completely with AcomA occlusion. **(E)** Panel 1: 3D-DSA showing an AcomA aneurysm (arrow); Panel 2: Roadmap image showing a microcatheter (arrowheads) crossing the stent into the contralateral ACA to wait to deploy another stent; Panel 3: X-ray image showing the “Y” configure stents, the arrowheads indicate the distal markers of the stent; Panel 4: Postoperative immediate DSA showing the aneurysm was coiled. **(F)** Left panel: 3D-DSA showing a sidewall AcomA aneurysm (arrow); Right panel: Unsubtracted DSA showing that the aneurysm was covered by FD deployment. **(G)** Left panel: DSA showing that the AcomA aneurysm with the WEB device in it; Right panel: Unsubtracted DSA showing the WEB device outline. 3D, three-dimensional; A1, first segment; ACA, anterior cerebral artery; AcomA, anterior communicating artery; AVM, arteriovenous malformation; CTA, computed tomography angiography; DSA, digital subtracted angiography; FD, flow diverter; PTA, primitive trigeminal artery; WEB, Woven EndoBridge.

EVT for AcomA aneurysms is challenging because of the small size, superior or posterior orientation of the aneurysm dome, presence of perforators and fenestration, and association with the AVM. EVT techniques include traditional coiling and deployment of FD and WEB devices. Traditional coiling with/without single or double “X- or Y-configuration” stenting or balloon assistance is still a well-established option (Figures 6A–E) (59). The AcomA may be compromised without serious complications during coiling; if A1 flows are symmetric, it is often sufficient to use a single stent to assist coiling (Figure 6D) (60).

FD treatment can be used for AcomA aneurysms (61). FDs should cover the AcomA aneurysm neck to the full extent by deployment from the A1 segment to the ipsilateral or contralateral A2 segment (62). Because AcomA aneurysm occlusion after FD treatment requires time, adjunctive coiling to prevent rebleeding is necessary for ruptured aneurysms (63). Not all AcomA aneurysms can achieve adequate occlusion after FD treatment. Occlusion depends on blood flow into the aneurysm. For sidewall aneurysms with thin or absent AcomA or aneurysms with contralateral hypoplastic or aplastic A1, FD treatment can cause adequate aneurysm occlusion (Figure 6F). For AcomA aneurysms with bilateral hyperplastic A1 segments, after FD deployment, adequate aneurysm occlusion is difficult because of the inefficacy of flow diversion (64). In these aneurysms, bilateral parallel “H configure” FD deployment in the ACA may be helpful for curing the aneurysm (62, 65).

For AcomA aneurysms, the WEB device’s broad base provides a stable construct and allows it to sit above the aneurysm neck (Figure 6G). The WEB device has the advantage of not requiring the use of antiplatelets. Some studies have demonstrated the safety and effectiveness of the WEB device for not only unruptured but also ruptured AcomA aneurysms, with low periprocedural morbidity and mortality (66, 67). In Adeeb et al.’s report, a total of 572 aneurysms were included; AcomA aneurysms accounted for 35.7%, and during the follow-up, the rate of adequate aneurysm occlusion was 80.6% (68). The WEB device should be applied selectively. AcomA aneurysms with a unilateral A1 are associated with better angiographic outcomes after WEB device treatment, perhaps because of less flow into the aneurysm (69). When the WEB device is deployed, technical complications, including access complications, vascular dissection, and deployment issues, must be considered (70). The deployment of the WEB device in AcomA aneurysms is associated with hemorrhagic and ischemic complications. In Adeeb et al.’s report, the rate of thromboembolic complications was 9.3% in AcomA aneurysms (68). In the CLinical Evaluation of the WEB 17 device in intracranial aneurysms (CLEVER), AcomA aneurysms accounted for 37.4%, and the rate of adverse events occurring on the day of the procedure was 11.7% (66). In addition to the WEB device, the Contour Neurovascular System (Cerus Endovascular, Fremont, CA), an intrasaccular device for the endovascular treatment of cerebral aneurysms, has shown safety and efficacy comparable to those of existing intrasaccular devices at the 1-year follow-up in a multicenter cohort study (71).

### 6.3 Distal aneurysms

**Figure 7:**
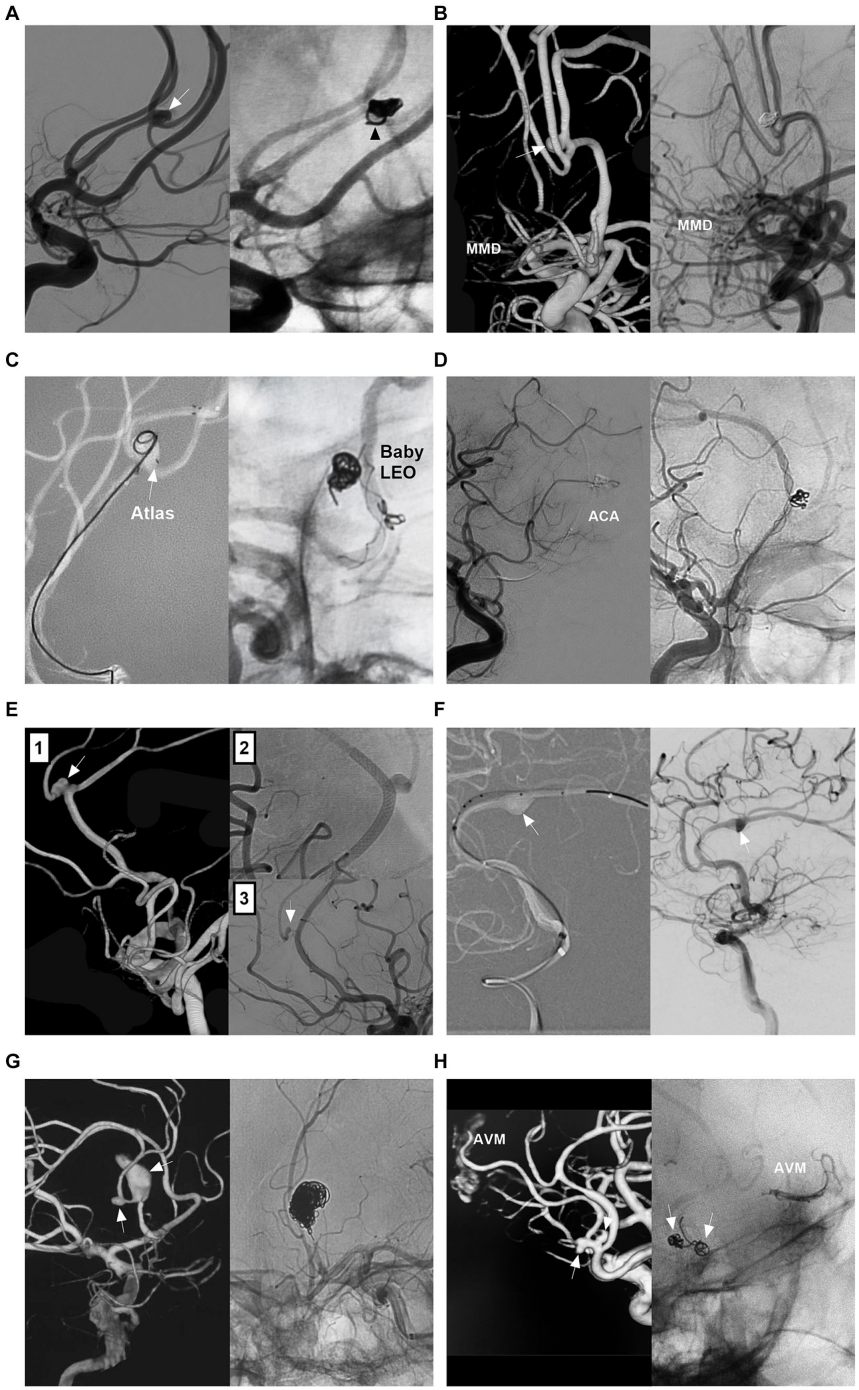
Treatment of distal ACA aneurysms. **(A)** Left panel: DSA showing a distal ACA aneurysm (arrow); Right panel: DSA showing that the aneurysm was coiled completely; one coil loop (arrowhead) provided support without stent assistance. **(B)** Left panel: 3D-DSA showing a distal ACA sidewall aneurysm (arrow) with MMD; Right panel: DSA showing that the aneurysm was coiled completely. **(C)** Left panel: Roadmap image showing a distal ACA wide-necked aneurysm (arrow) was coiled with Atlas stent assistance, the tail of the stent (arrow) was deployed in the aneurysm; Right panel: Unsubtracted DSA showing a small ACA aneurysm above the AcomA was coiled with baby LEO stent assistance. **(D)** Left panel: DSA showing the occlusion of the ACA after the catheterization of double microcatheters; Right panel: Unsubtracted DSA showing the ACA recanalization after removing the microcatheters. **(E)** Panel 1: 3D-DSA showing a distal ACA aneurysm with a branch incorporation (arrow); Panel 2: panel: DSA showing the flow diverter covering the aneurysm; Panel 3: Follow-up DSA showing the aneurysm regressed. **(F)** Left panel: Roadmap image showing that a distal ACA dissecting aneurysm (arrow) was being covered by a flow diverter; Right panel: DSA showing the contrast agent retention (arrow) in the aneurysm. **(G)** Left panel: 3D-DSA showing two tandem A2 aneurysms (arrows); Right panel: Unsubtracted DSA showing that the proximal large aneurysm together with proximal parent artery was occluded. **(H)** Left panel: 3D-DSA showing two tandem aneurysms (arrows) at the origin of the feeding artery to an AVM; Right panel: Unsubtracted DSA showing that the aneurysms (arrows) were coiled, and the AVM was embolized by Onyx. 3D, three-dimensional; ACA, anterior cerebral artery; AVM, arteriovenous malformation; CTA, computed tomography angiography; DSA, digital subtracted angiography; MMD, moyamoya disease.

Distal ACA aneurysms often have a small size with a narrow parent artery, a wide neck with branch incorporation, and a distal location with a tortuous approach (Figures 7A,B), which increases the difficulty of EVT, and EVT is often associated with high risks of arterial dissection, intraprocedural rupture, inadvertent parent vessel occlusion, incomplete aneurysm occlusion, etc. (23). For distal ACA aneurysms that necessitate double microcatheterization for stent-assisted coiling or balloon remodeling, dual microcatheter manipulation in small arteries can be feasible but challenging (Figures 7C,D).

FDs can be used for distal ACA aneurysms (72). For FDs, adequate sizing to achieve adequate wall apposition to the parent wall to prevent endoleak is imperative. The ACA diameter is approximately 2 mm. The FD diameter may range between 2 mm and 2.75 mm (72). Although FD oversizing can result in subsequent FD elongation and a relative reduction in metal coverage, the flow diversion effect is sufficient to allow high rates of distal ACA aneurysm occlusion. For FD treatment for distal ACA aneurysms, there are important concerns regarding possible occlusion of the covered branch (73). Theoretically, after FD deployment, the pressure gradient across the covered branch is reduced, and the branch can be occluded if flow competition from the collateral circulation is well represented (Figures 7E,F) (72, 74). However, acute occlusion of the branch can occur, resulting in ischemia.

For distal ACA aneurysms that require difficult reconstructive treatment, such as traumatic, infectious, flow-related, giant or serpentine aneurysms, PAO can be considered (Figures 7G,H) (5, 75, 76).

### 6.4 Antiplatelet management in FD deployment

On the basis of increasing experience deploying FDs in the last decade, many practitioners have become accustomed to using dual antiplatelet therapy (DAPT). However, the management of FD deployment with antiplatelet agents is heterogeneous and varies in terms of drug type, dose and medication time. The traditional DAPT regimen consists of aspirin and clopidogrel. However, standard daily oral doses of clopidogrel fail to completely inhibit adenosine diphosphate-induced platelet aggregation in up to 30% of patients, a phenomenon referred to as a ‘poor response’ (77). Many practitioners use ticagrelor or prasugrel instead of clopidogrel. Podlasek et al.’s meta-analysis with 1,005 patients undergoing FD deployment concluded that DAPT regimens, including ticagrelor or prasugrel, are safe and that the use of ticagrelor may be associated with better survival than the use of clopidogrel (78).

The following suggestions for antiplatelet management from our institute can be referred to. If patients were identified as clopidogrel nonresponders by platelet function testing, DAPT (aspirin and ticagrelor) was given. For unruptured aneurysms, DAPT was given for 5–7 days before EVT. For ruptured aneurysms, before at least three hours of EVT, a loading dose of DAPT was given. From the second day of EVT, for all patients, DAPT was continued for six months. Then, follow-up angiography was performed. According to the follow-up angiography results, if the aneurysm was occluded and the FD was patent, DAPT was transitioned to single antiplatelet treatment with aspirin that was continued for six months to one year or life, depending on the patient’s clinical and radiological circumstances.

## 7 Prognosis and complications

For EVT for ACA aneurysms, a good clinical outcome is defined as a modified Rankin scale (mRS) score of ≤2 or a Glasgow Outcome Scale (GOS) score of 4 or 5 (23, 79). Adequate angiographic aneurysm occlusion can be assessed by the Raymond–Roy (RR) occlusion scale and classified as class 1 (complete occlusion) or class 2 (neck residual <2 mm) (80). Adequate angiographic aneurysm occlusion can also be divided into complete occlusion (no aneurysm visualization) and nearly complete occlusion (the presence of a small residual neck) (52). Traditional coiling, FD deployment and WEB can also use the above classifications to assess angiographic aneurysm occlusion.

For FD deployment, treatment outcomes can be graded by the O’Kelly Marotta (OKM) grading scale; on this scale, the degree of filling (A = total, B = subtotal, C = entry remnant, D = no filling) and the degree of stasis (prolongation of stasis into 1 = arterial, 2 = capillary, or 3 = venous phase) are rated (81). Adequate angiographic aneurysm outcomes include C-D degrees of filling and 2–3 degrees of stasis.

### 7.1 Proximal ACA aneurysms

**Figure 8:**
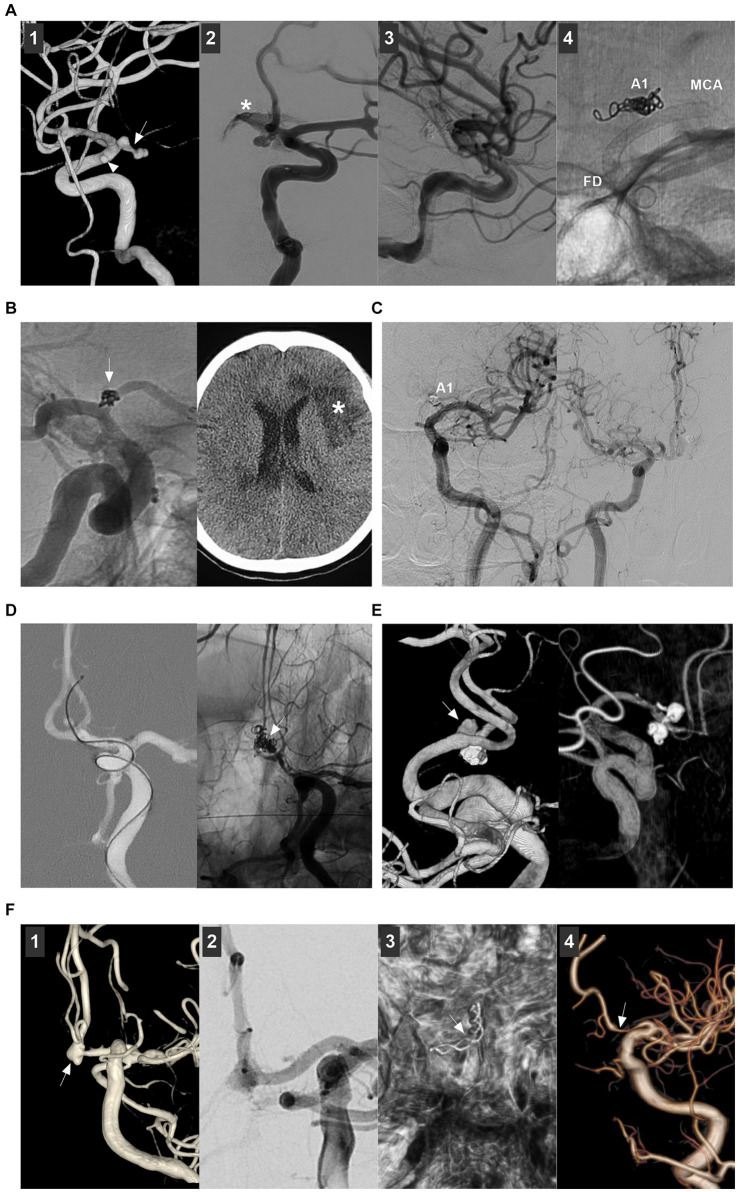
Complications of endovascular treatment for proximal ACA and AcomA aneurysms. **(A)** Panel 1: 3D-DSA showing an A1 aneurysm (arrow) and an anterior choroidal aneurysm (arrowhead); Panel 2: DSA showing the aneurysm rupture during the catheterization, the asterisk indicated contrast agent extravasation; Panel 3: DSA showing that the bleeding stopped by continuous coiling; Panel 4: X ray film showing the coiling in A1 segment origin, the flow diverter covered the A1 origin to avoid the aneurysm rerupture. **(B)** Left panel: DSA showing that the A1 aneurysm (arrow) was coiled; Right panel: Postoperative CT showing the ischemia (asterisk). **(C)** Left panel: Follow-up DSA showing that the asymptomatic occlusion of A1 segment after coiling the A1 aneurysm; Right panel: Follow-up DSA showing that A1 segment had sufficient blood flow from contralateral ACA. **(D)** Roadmap DSA showing that the AcomA aneurysm was perforated by the microcatheter (arrow); Right panel: Unsubtracted DSA showing the aneurysm was coiled (arrow). **(E)** Left panel: 3D-DSA showing a *de novo* AcomA aneurysm (arrow); the previous coiled aneurysm was below. Right panel: Six-month follow-up 3D-DSA showing that the aneurysm had no recurrence. **(F)** Panel 1: 3D-DSA showing a AcomA aneurysm (arrow); Panel 2: DSA showing the AcomA aneurysm was covered by flow diverter; Panel 3: Xper-CT showing the flow diverter (arrow); Panel 4: Six-month follow-up DSA showing that the aneurysm was cured and the proximal ACA was stenotic (arrow). 3D, three-dimensional; ACA, anterior cerebral artery; AcomA, anterior communicating aneurysm; CT, computed tomography; DSA, digital subtracted angiography.

Although traditional EVT for A1 aneurysms is difficult, it still has a > 80% rate of good clinical outcomes and adequate angiographic outcomes, as shown in Zhang et al.’s report on 32 A1 aneurysms (82), Kwon et al.’s report on 11 A1 aneurysms (54), Kim et al.’s report on 20 A1 aneurysms (83), and Li et al.’s report on 15 A1 aneurysms (84). However, there were procedure-related complications (Figures 8A–C). For example, the rate of intraoperative rupture was 3.1% in Zhang et al.’s report (82) and 6% in Li et al.’s report (84). The rate of ischemic complications was 9.1% in Kwon et al.’s report (54). Some A1 aneurysms can suffer recurrence after EVT. The recurrence rate was 3.1% in Zhang et al.’s report (82) and 13.3% in Li et al.’s report (84). According to limited data from case reports, deconstructive PAO and FD treatment for giant, fusiform, and dissecting A1 aneurysms also results in good outcomes (56, 58, 85, 86).

### 7.2 AcomA aneurysms

EVT for AcomA aneurysms can result in a > 85% rate of good clinical and adequate angiographic outcomes (27, 50, 61, 62, 64, 87). However, EVT for AcomA aneurysms may be associated with procedure-related complications (Figures 8D–F). In Yarahmadi et al.’s meta-analysis, the overall rate of procedure-related complications of EVT for AcomA aneurysms was 9.6%, of which the rates of thromboembolic events, intraoperative rupture, coil prolapse and postoperative early rebleeding were 6.1, 4.2, 4.7, and 2.2%, respectively (88). In Diana et al.’s meta-analysis, FD treatment, stent-assisted coiling and endoscopic devices caused minor complications in 11.8, 3.8, and 14.3% of cases, respectively, and major complications in 3.2, 4.4, and 0% of cases, respectively (50).

Ischemic events can be caused by occlusion of the parent artery or branch or perforating artery and in-stent thrombosis. For ischemic events, mechanical thrombectomy, intravenous administration of tirofiban, and clot maceration with microcatheters and guidewires can be used. Hemorrhagic events included intraoperative and delayed aneurysm rupture. Intraoperative aneurysm rupture is believed to be due to the increased restriction of microcatheter movement within small aneurysms. AcomA aneurysms <4 mm in size have a 5-fold greater incidence of intraoperative rupture during coiling (89). Intraoperative AcomA aneurysm perforations can be controlled by continuous coiling. Postoperative delayed aneurysm rupture or parent artery stenosis can occur after FD deployment (Figure 8F). Postoperative aneurysm rupture may occur in large or giant AcomA aneurysms because of the increased pressure in the aneurysm sac caused by malignancy (90). To date, there are no effective measures to prevent this type of complication, although double FDs and adjunctive coiling may be helpful (90). Parent artery stenosis after FD deployment is often a benign and self-limiting complication (91).

After EVT, AcomA aneurysms may recur and require further treatment (Figure 8E). In Sattari et al.’s meta-analysis, the rates of recurrence and retreatment after EVT for AcomA aneurysms were 10.8 and 3.5%, respectively (21). In Catapano et al.’s report, the rates were 13.4 and 9.7%, respectively (92). Many factors can be associated with the EVT-related recurrence of AcomA aneurysms. Large AcomA aneurysms >7 mm in size and RR occlusion scale class III are strong risk factors (92, 93). After recurrence and retreatment, the rebleeding rate is very low, which implies that, regardless of the recurrence or retreatment status, EVT for AcomA aneurysms is safe (61, 92, 94).

Using the WEB device for AcomA aneurysms can result in an acceptable outcome. In Adeeb et al.’s report, AcomA aneurysms had an adequate occlusion rate of 80.6% after WEB device deployment (68). In an international multicenter study, the adequate occlusion rate was 76.2% for ruptured AcomA aneurysms treated with a WEB device, and there was no significant difference in either the radiologic outcomes or complications between unruptured and ruptured aneurysms (70).

### 7.3 Distal ACA aneurysms

EVT techniques for distal ACA aneurysms are associated with a nearly 80% rate of good clinical outcomes and a nearly 70% rate of adequate angiographic outcomes (23, 95). In recent years, with the development of EVT techniques, EVT outcomes have improved. In Porto et al.’s study, during the final follow-up, EVT resulted in an 85.5% rate of good clinical outcome and 95.3 and 89.5% rates of adequate aneurysm occlusion in traditional coiling and FD treatment, respectively (96). In the report by Metayer et al., during a 1-year follow-up, EVT resulted in an 81.2% rate of good clinical outcome and a 96.2% rate of adequate aneurysm occlusion treated with traditional coiling and FD (18). In addition, owing to the limited data from case reports, stent-assisted coiling, FD deployment and PAO can result in good outcomes for nonsaccular ACA aneurysms (97, 98).

**Figure 9:**
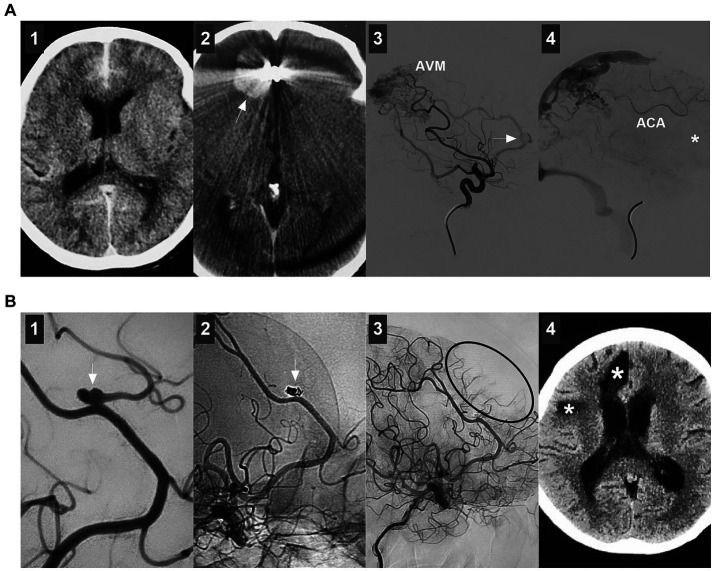
Complications of endovascular treatment for distal ACA aneurysms. **(A)** Panel 1: CT showing subarachnoid hemorrhage; Panel 2: Postoperative one-week CT showing a hematoma (arrow) in right frontal lobe; Panel 3: DSA showing that the flow-related aneurysm (arrow) on the ACA was incompletely coiled under the stenting assistance; Panel 4: DSA showing that the flow-related aneurysm (arrow) was incompletely coiled by parent artery occlusion at the asterisk location, the retrograde flow of ACA was sufficient. **(B)** Panel 1: DSA showing a distal ACA aneurysm (arrow); Panel 2: Unsubtracted DSA showing that the aneurysm (arrow) was coiled, and the branch from the aneurysm was occluded; Panel 3: DSA showing the insufficient blood flow by the occluded branch (circle); Panel 4: Six-month follow-up CT showing encephalomalacia (asterisks). ACA, anterior cerebral artery; AVM, arteriovenous malformation; CT, computed tomography; DSA, digital subtracted angiography.

However, EVT for distal ACA aneurysms is still associated with high rates of complications and recurrence (Figure 9). The rates of procedure-related complications were reported to be 12% in Sturiale et al.’s systematic review (52), 13% in Porto et al.’s report (96), and 7.6% in Liao et al.’s report (95). The rates of recurrence were 19.1% in Petr et al.’s meta-analysis (23), 15% in Metayer et al.’s report (18), and 10.2% in Park et al.’s report (99). The wide angle between 2 distal branches of the distal ACA aneurysm and its irregular shape might be independent risk factors for recurrence after EVT for distal ACA aneurysms (100). FD treatment helps reduce recurrence. In Cagnazzo et al.’s report, no retreatment was reported after FD deployment (73).

## 8 Summary

ACA aneurysms are complex, and EVT is becoming an alternative treatment for ACA aneurysms. EVT techniques for ACA aneurysms vary according to the nature and location of the aneurysm. Currently, traditional coiling is still the preferred treatment for most ACA aneurysms. However, for A1 aneurysms, EVT is challenging, and it can only be used in select A1 aneurysms. After the selection of appropriate cases, FD deployment can result in a good prognosis for ACA aneurysms. In addition, PAO can be used to treat A1 aneurysms with good collateral circulation and some distal ACA aneurysms. In general, EVT is gaining popularity as an alternative treatment option for ACA aneurysms (Table 1).

**Table 1:** EVT status for ACA aneurysms.

Aneurysm location	EVT status
A1	Coiling can only be performed in A1 aneurysms with easy catheterization, such as those located at the beginning and termination of A1 segment. In selective cases, a contralateral approach via the AcomA can be used to coil A1 aneurysms if a competent AcomA exists. For selective nonsaccular aneurysms, PAO and FD deployment can be performed.
AcomA	Coiling AcomA aneurysms is still a well-established treatment option. For wide-necked AcomA aneurysms, stenting can be used to assist the coiling. For sidewall unruptured aneurysms with thin or absent AcomA, FD treatment was feasible. In selective AcomA aneurysms with the easy catheterization, the WEB device can be used.
Distal ACA	For distal ACA aneurysms, dual microcatheter manipulation for stent-assisted coiling can be feasible, but challenging. FD can be used for distal ACA unruptured aneurysms. For distal ACA aneurysms that require difficult reconstructive treatment, PAO can be considered after evaluating collateral circulation.

A1, First segment; ACA, anterior cerebral artery; AcomA, anterior communicating artery; EVT, endovascular treatment; FD, flow diverter; PAO, parent artery occlusion; WEB, Woven EndoBridge.

## Author contributions

BL: Writing – original draft, Data curation. KZ: Data curation, Writing – original draft. JY: Conceptualization, Writing – original draft, Writing – review & editing.
